# DK1 Induces Apoptosis via Mitochondria-Dependent Signaling Pathway in Human Colon Carcinoma Cell Lines In Vitro

**DOI:** 10.3390/ijms19041151

**Published:** 2018-04-11

**Authors:** Yazmin Hussin, Muhammad Nazirul Mubin Aziz, Nurul Fattin Che Rahim, Swee Keong Yeap, Nurul Elyani Mohamad, Mas Jaffri Masarudin, Noraini Nordin, Nik Mohd Afizan-Nik Abd Rahman, Chean Yeah Yong, Muhammad Nadeem Akhtar, Siti Noor Hajar Zamrus, Noorjahan Banu Alitheen

**Affiliations:** 1 Department of Cell and Molecular Biology, Faculty of Biotechnology and Biomolecular Sciences, Universiti Putra Malaysia, Serdang 43400, Malaysia; yazminh93@gmail.com (Y.H.); muhammadnazirulmubin@gmail.com (M.N.M.A.); nurulfattincherahim@gmail.com (N.F.C.R.); elyani.mohamad@gmail.com (N.E.M.); masjaffri@upm.edu.my (M.J.M.); noraininordin1303@gmail.com (N.N.); m.afizan@upm.edu.my (N.M.A.-N.A.R.); 2 China-ASEAN College of Marine Science, Xiamen University Malaysia, Sepang 43900, Malaysia; skyeap2005@gmail.com; 3 Department of Microbiology, Faculty of Biotechnology and Biomolecular Sciences, Universiti Putra Malaysia, Serdang 43400, Malaysia; yongcheanyeah@gmail.com; 4 Faculty of Industrial Sciences and Technology, Universiti Malaysia Pahang, Kuantan 26300, Malaysia; nadeemupm@gmail.com (M.N.A.); hajar202703@gmail.com (S.N.H.Z.)

**Keywords:** DK1, humancolon cancer, curcumin analog, HT29, SW620

## Abstract

Extensive research has been done in the search for innovative treatments against colon adenocarcinomas; however, the incidence rate of patients remains a major cause of cancer-related deaths in Malaysia. Natural bioactive compounds such as curcumin have been substantially studied as an alternative to anticancer drug therapies and have been surmised as a potent agent but, nevertheless, remain deficient due to its poor cellular uptake. Therefore, efforts now have shifted toward mimicking curcumin to synthesize novel compounds sharing similar effects. A synthetic analog, (*Z*)-3-hydroxy-1-(2-hydroxyphenyl)-3-phenylprop-2-ene-1-one (DK1), was recently synthesized and reported to confer improved bioavailability and selectivity toward human breast cancer cells. This study, therefore, aims to assess the anticancer mechanism of DK1 in relation to the induction of in vitro cell death in selected human colon cancer cell lines. Using the3-(4,5-dimethylthiazol-2-yl)-2,5-diphenyltetrazolium bromide(MTT) assay, the cytotoxicity of DK1 towards HT29 and SW620 cell lines were investigated. Acridine orange/propidium iodide (AO/PI) dual-staining assay and flow cytometry analyses (cell cycle analysis, Annexin/V-FITC and JC-1 assays) were incorporated to determine the mode of cell death. To further determine the mechanism of cell death, quantitative real-time polymerase chain reaction (qRT-PCR) and proteome profiling were conducted. Results from this study suggest that DK1 induced changes in cell morphology, leading to a decrease in cell viability and subsequent induction of apoptosis. DK1 treatment inhibited cell viability and proliferation 48 h post treatment with IC_50_ values of 7.5 ± 1.6 µM for HT29 cells and 14.5 ± 4.3 µM for SW620 cells, causing cell cycle arrest with increased accumulation of cell populations at the sub-G_0_/G_1_phaseof 74% and 23%, respectively. Flow cytometry analyses showed that DK1 treatment in cancer cells induced apoptosis, as indicated by DNA fragmentation and depolarization of the mitochondrial membrane. qRT-PCR results show significant upregulation in the expression of caspase-9 in both HT29 and SW620 cell lines, further supporting that cell death induction by DK1 is via an intrinsic pathway. These outcomes, therefore, demonstrate DK1 as a potential anticancer agent for colon adenocarcinoma due to its anti-apoptotic attributes.

## 1. Introduction

Cancer remains a pathological hurdle that mankind has continuously tried to overcome for many years. Currently, colon cancer is the second leading cause of death of cancer globally, trailing lung cancer. In most countries, the rate of colorectal incidence increases in parallel to the increment in human development index (HDI) levels; however, it was concluded that the rates tend to alleviate and then progressively decrease in countries with very high HDI levels [1]. The HDI level is an index measurement of human development and how it progresses with time, which is a composite index for three dimensions of human development: longevity, education, and standard of living [1]. An increasing number of studies have found that the factors contributing to a higher incidence of colon cancer in men may be in part due to irregular activities of the male hormone testosterone, which indirectly promotes early adenomagenesis [2]. Common treatments currently practiced for colon cancer include colectomy, radiotherapy, and chemotherapy, which often leads to permanent side effects in treated patients. Permanent side effects may also be contributed by the need to perform colonoscopies every 1–2 years for the removal of precancerous polyps [3]. This inconvenience and the highly invasive treatment routes, therefore, warrant efforts for alternative treatment with fewer side effects for colon cancer patients.

Among the popular alternatives being extensively studied is the administration of natural compounds for anticancer therapy, such as curcumin, also known as diferuloylmethane, which is a member of the curcuminoid family. Curcumin is the main active compound found in *Curcuma longa*, more commonly known as turmeric, and is used widely by Malaysian locals as a food additive and a cosmetic ingredient. Over the years, studies have shown that curcumin possesses many pharmacological characteristics: neuroprotective [4,5], antioxidant [6], antiinflammatory [7] and antifungal [8], immunomodulatory [9], and antimalarial [6,10] activities. However, curcumin has poor aqueous solubility and, therefore, much research has been conducted in developing synthetic compounds with better solubility.

**Figure 1:**
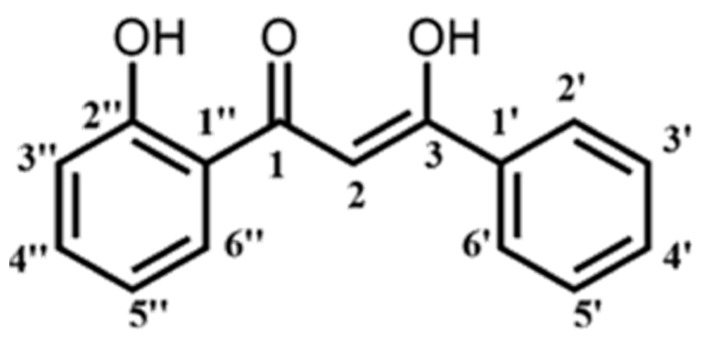
The structure of (*Z*)-3-hydroxy-1-(2-hydroxyphenyl)-3-phenylprop-2-ene-1-one (DK1) as reported by Ali et al. (2017) [15].

Synthetic compounds such as isoform derivatives and analogs are commonly synthesized to overcome the weakness and limitations possessed by its parent compound. Many studies have synthesized curcumin analogs to improve its anticancer properties [11,12,13,14,15]. Recently, a synthetic analog of curcumin possessing a lower molecular weight—namely, (*Z*)-3-hydroxy-1-(2-hydroxyphenyl)-3-phenylprop-2-ene-1-one (DK1)—was synthesized in the form of a 100% pure crystal via the Baker–Venkataraman rearrangement method and confirmed using single X-ray crystallography [15]. A recent study by Ali et al. found that DK1 treatment was more selective and cytotoxic towards MCF-7 and MDA MB-213 breast cancer cell lines thantheMCF-10A normal breast cell line and induced G2/M phase arrest in the breast cancer cells [15]. Aziz et al. reported remarkable results where DK1 induced S phase arrest in cell cycle progression and apoptosis via the intrinsic pathway in two bone cancer cell lines, U-2 OS and MG-63 [16]. These two studies showed that DK1 may be a potential multi-target drug in cancer therapy. Therefore, this study aimed to assess the potential of DK1 as a multi-target drug in cancer therapy and also to assess whether the anticancer potential ofDK1(Figure 1) ((*Z*)-3-hydroxy-1-(2-hydroxyphenyl)-3-phenylprop-2-ene-1-one) could be extended to different cancer types, such as colon adenocarcinoma, by investigating the anticancer effect and the mechanism of DK1-mediated cell death induction in human colon cancer cells (HT29 and SW620) in vitro. Our results suggest that DK1 possesses better anticancer potential than curcumin and an ability to induce apoptosis in colorectal adenocarcinoma cell lines.

## 2. Results

### 2.1. DK1 Decreases Cell Viability of HT29 and SW620

The MTT assay was used as a preliminary assessment of the anti-proliferative effects of natural curcumin and DK1 against two human colon cancer cell lines—HT29 and SW620. This assay was performed to measure the inhibitory concentrations that would kill 25%, 50%, and 75% of the total cell population, defined as the IC_25_, IC_50_, and IC_75_, respectively. Cells were treated with two-fold serial dilutions of the natural curcumin compound and its analog DK1 and incubated for 48 h. Table 1 shows the inhibitory concentrations of curcumin and DK1 against HT29 and SW620 cells 48 h post treatment. As depicted in Table 1, the IC_25_ and IC_50_ values of DK1 in HT29 cells (2.8 µM and 7.5 µM, respectively) were lower than in SW620 cells (3.7 µM and 14.5 µM, respectively). However, the IC_75_ values of DK1 were found to be similar between both cell lines. It was noted that within 48 h post incubation, the IC_50_ of curcumin was much higher compared to DK1 for both HT29 and SW620 cell lines. These inhibitory concentrations were subsequently used for the following assays conducted in this study.

### 2.2. Treatment of DK1 Alters Morphological Appearance and Induces Apoptosis in HT29 and SW620 

**Figure 2:**
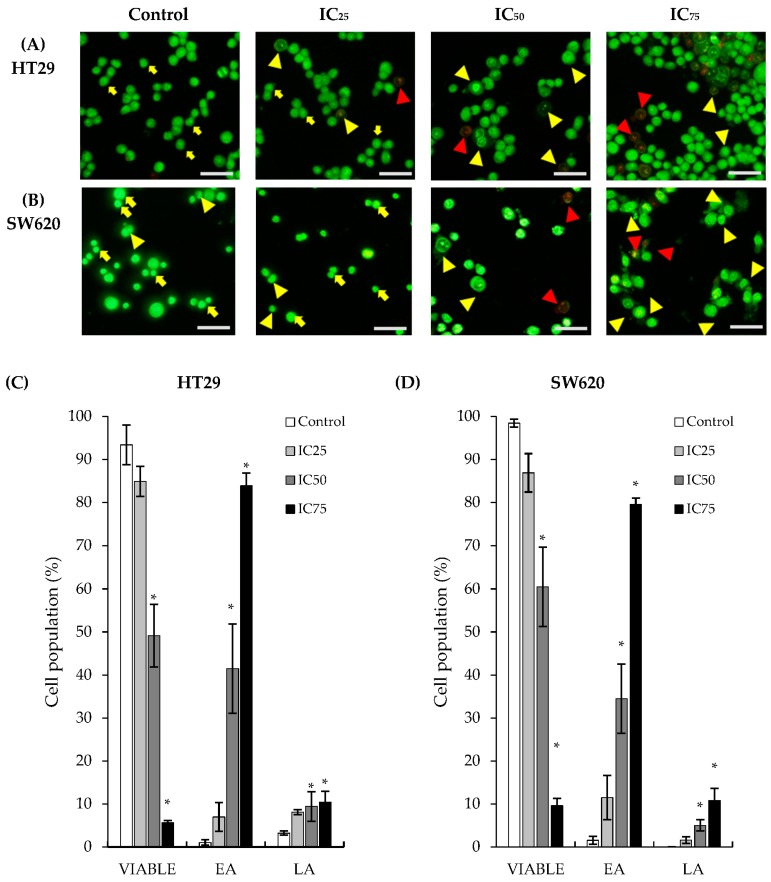
Representative images of morphological changes in (**A**) HT29 and (**B**) SW620 after 48 h of DK1 treatment (yellow arrow: viable; yellow arrowhead: early apoptotic cells; red arrowhead: late apoptotic cells). Cell viability quantification of (**C**) HT29 and (**D**) SW620 cells after48 h of DK1 treatment in a population of 200 cells. EA represents early apoptosis and LA represents late apoptosis. All data are expressed as mean ± SD.* *p >* 0.05 compared to corresponding controls. (Magnification of objective: 20×; scale bar represents 2000 µm).

The acridine orange/propidium iodide (AO/PI) dual-staining assay was carried out to observe morphological changes in treated cells and to semi-quantify the viable, apoptotic, and necrotic cells after DK1 treatments, thus elucidating the mode of cell death caused by the treatment. Viable cells were cells that fluoresced green with an intact cell membrane; early apoptotic cells were characterized by the occurrences of membrane blebbing and nuclear condensation whilst still fluorescing green; late apoptotic cells were characterized by a compromised membrane, nuclear distortion, and yellow-orange fluorescence with apoptotic bodies; and necrotic cells were cells that fluoresced red [15]. As depicted in Figure 2, the number of viable cells decreased with dosage of DK1 treatment, suggesting that DK1 induced apoptosis in both cell lines dosedependently. Cells were observed to begin displaying characteristics that indicated apoptosis in IC_25_ treatments of both cell lines, evidently more prominent in HT29 cells than in SW620 cells. As the DK1 dosage increased to the IC_50_, membrane blebbing occurred and the number of apoptotic cells increased. At the highest IC_75_ dose, the most significant increase in apoptotic populations of both HT29 and SW620 cells was exhibited. The cells were seen to undergo cell shrinkage.

**Figure 3:**
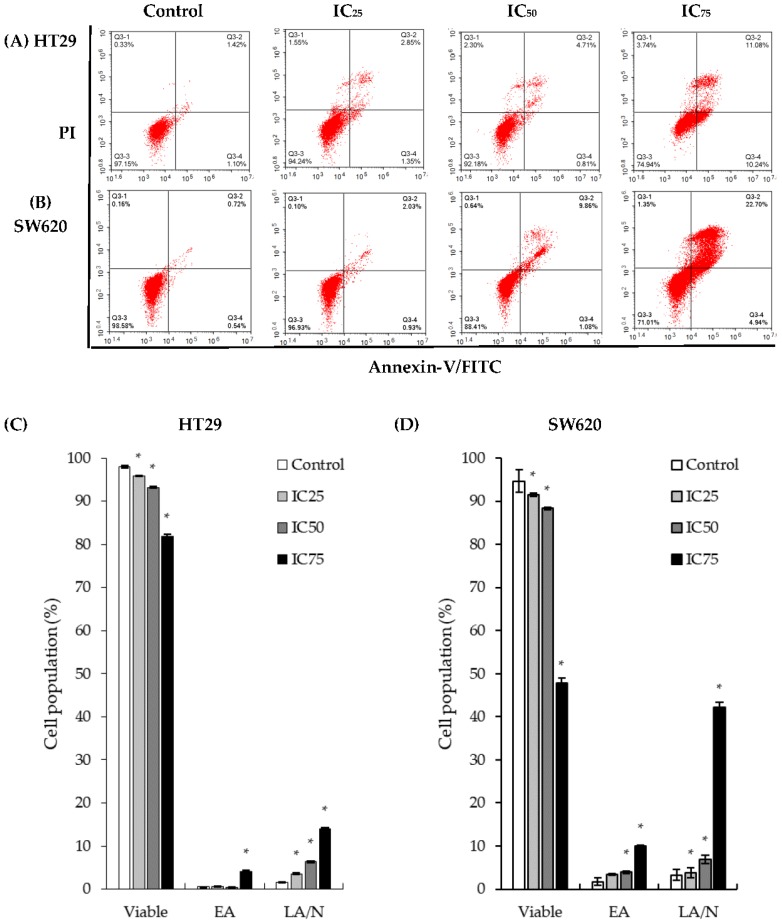
Flow cytometry annexin-V/FITC analysis.Representative histogram analyses of annexin-V/FITC assay after 48 h of three concentrations of DK1 treatment (IC_25_, IC_50_, and IC_75_)of (**A**) HT29 and (**B**) SW620 cells. Quantification analysis of annexin-V/FITC analysis of (**C**) HT29 and (**D**) SW620 cells after 48 h of DK1 treatment. EA represents early apoptosis, while LA/N represents late apoptosis and necrosis.All data are expressed as mean ± SD. * *p* < 0.05 compared with corresponding controls.

Additionally, as shown in Figure 3, apoptotic cell populations in both cell lines showed a significant increase when treated with DK1. The viable HT29 cell population decreased from 96% in untreated control cells to 80% in IC_50_treatment of DK1; while in SW620 cells the viable cell population showed a slight decrease from 98% in untreated control cells to 88% in the IC_50_ treatment. However, a pronounced increase in the annexin-V+/PI+ quadrant, indicating late apoptosis, was detected from 1% of the cell population in control cells to 2% in IC_25_ treatment, 4% in IC_50_ and finally 11% in IC_75_ treatments of DK1. SW620 cells also displayed a steady late apoptotic population increase from 2% in IC_25_ treatments of DK1 to approximately 10% in IC_50_treatments and 22% in IC_75_ treatments.

### 2.3. Cell Cycle Arrest at G2/M Phase in HT29 and SW620 Cells

**Figure 4:**
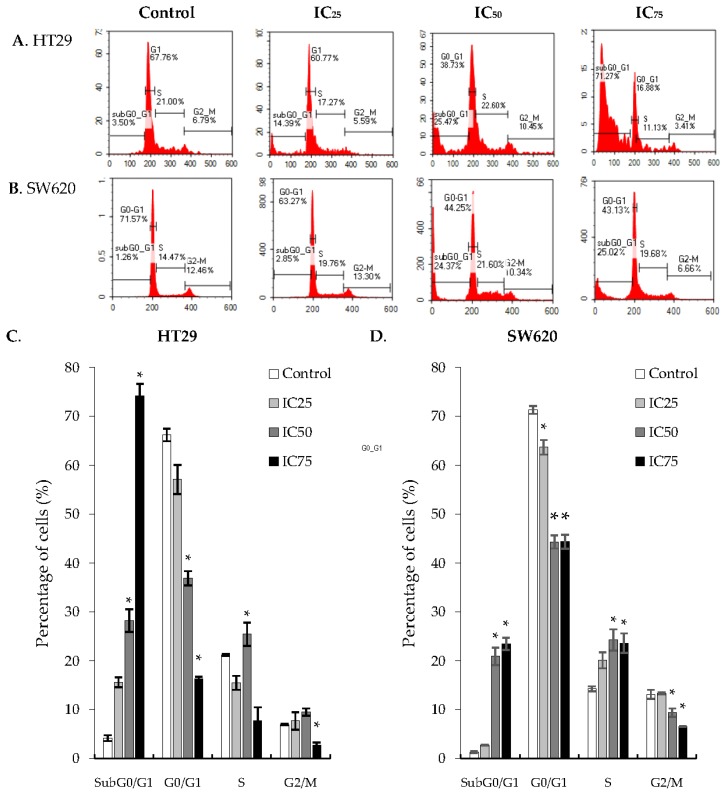
Cell cycle analysis. Quantification of cell cycle analysis of (**A**) HT29 and (**B**) SW620 cells after 48 h of DK1 treatment (IC_25_, IC_50_, and IC_75_). Quantification analyses of the cell cycle analysis of (**C**) HT29 and (**D**) SW620 cells after 48 h of three concentrations of DK1. All data are expressed as mean ± SD. * *p* < 0.05 compared with corresponding controls.

Cancer cells have irregular cell cycle progression profiles due to the presence of growth factors and its inherent mutagenic nature. One favorable characteristic when formulating candidate compounds for cancer therapeutics is its ability to terminate the cell cycle at certain checkpoints, causing the treated cancer cells to be sensitized to damage. To further examine the effects of DK1 on the induction of apoptosis, its effects on the cell cycle was investigated. Cell cycle analysis was done using flow cytometry with PI to stain cellular DNA. Figure 4 shows the gradual increase in the sub-G_0_/G_1_ population of treated HT29 cells, from 4% in the untreated control group to 15%, 28%, and 74% when exposed to three different DK1concentrations (IC_25_, IC_50_,and IC_75_, respectively) for 48 h. In the treatment of SW620 cells, the sub-G_0_/G_1_ population increased to 20% and 23% when exposed to IC_50_ and IC_75_ concentrations of DK1 for 48 h. Cell cycle arrest for both cell lines occurred at the S phase based on a significant increase in the S phase populations when treated with DK1.

### 2.4. Apoptosis via Mitochondria-Dependent Pathway Induced by DK1 Treatment

**Figure 5:**
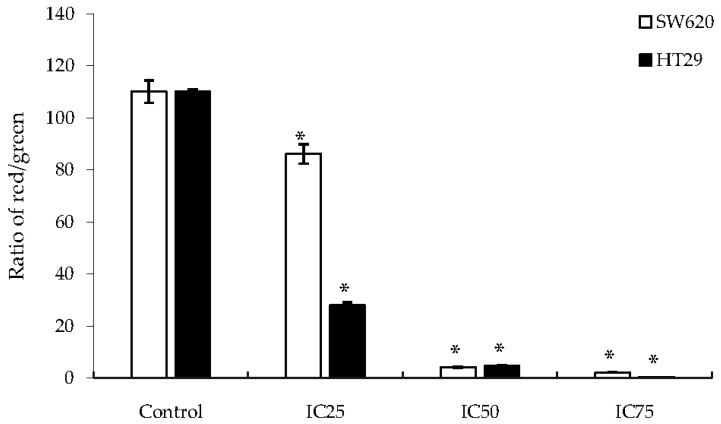
Depolarization of mitochondrial membrane potential. Quantification analyses of the JC-1 assay forHT29 and SW620 cells after 48 h of DK1 treatment showing the ratio of red to green fluorescence. All data are expressed as mean ± standard deviation (SD). * *p* < 0.05 compared with corresponding controls.

HT29 and SW620 cells were exposed to the JC-1 dye to measure their mitochondrial membrane potential (Δψ_M_). The permeabilization of the mitochondrial membrane plays an essential role in mitochondria-dependent apoptosis. Depolarization of the mitochondrial membrane induces the formation of the mitochondrial permeability transition pore, which activates the release of small molecules including pro-apoptotic factors, such as cytochrome c, into the cytosol [17]. The JC-1 dye exists in two forms: J-aggregates that fluoresce red when cells are healthy and the mitochondrial membrane potential is high, and J-monomers (its monomeric form) that emit green fluorescence and exist when the mitochondrial membrane potential is low. The ratio of red to green fluorescence depicts the strength of the mitochondrial membrane potential. Thus, healthy cells will confer a higher ratio as there would be a greater population of J-aggregates detected as compared to J-monomers. As shown in Figure 5, the ratio of aggregates to monomers decreased as a higher dosage of DK1 was administered, indicating that apoptosis was dosedependent.

### 2.5. DK1 Regulates Several Apoptotic Genes and Proteins

**Figure 6:**
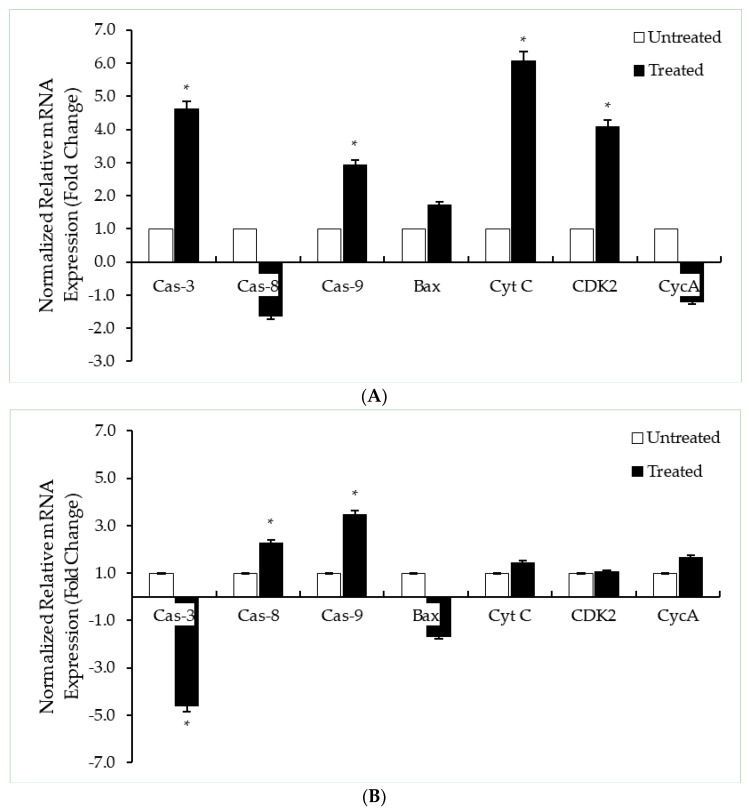
qRT-PCR analyses of apoptosis and cell cycle-related genes; caspase-3 (Cas-3), caspase-8 (cas-8), caspase-9 (cas-9), Bax, cytochrome C (Cyt C), Cdk 2 (CDK2), and cyclin A (CycA) for (**A**) HT29 and (**B**) SW620 cells when treated with DK1 (IC_50_) for 48 h. The expressions of the target genes were normalized to ACTB and 18S rRNA. All data are expressed as mean ± SD. * *p* < 0.05 compared to corresponding controls. The significance for this assay was set at >2-fold changes, comparing between the control (untreated) group and the treated group.

There are two main pathways of apoptosis: extrinsic and intrinsic. In order to confirm the pathway involved in the DK1-mediated cell death in the HT29 and SW620 cells, the transcriptome of the cells was further investigated. The effects of DK1 treatment towards HT29 and SW620 cells were further assessed by conducting qRT-PCR and human apoptosis proteome profiler. Apoptosis is mainly regulated by the caspase cascade and a family of apoptotic proteins called Bcl-2 family protein [16,18]. Caspase-3, caspase-8, and caspase-9 were chosen as these genes are involved in the caspase cascade that would then trigger the regulation of Bax and cytochrome c. The gene regulation of cyclin A and Cdk 2 genes were also assessed as they form a complex that plays an important role in the cell cycle progression at S phase, as shown in Figure 4. There was an increase in the expressions of caspase-3, caspase-9, cytochrome C, and Cdk 2 genes in HT29 cells compared to SW620 cells (Figure 6). After 48 h of DK1 (IC_50_) treatment, the expression levels of human pro-apoptosis proteins such as pro-caspase-3, cleaved caspase-3, Bax, cytochrome C, HTRA2/Omi, and SMAC/Diablo proteins were observed to be upregulated in HT29, while in SW620, the expressions decreased except for the increased HTRA2/Omi protein (Table 2).

## 3. Discussion

The synthesis of curcumin analogs often aims to increase the potential of commercially-ready dietary curcumin. Several studies have reported better selectivity and more effective anticancer potentials using chemically synthesized curcumin analogs as treatments [13,14]. It was reported that several chemically synthesized curcumin analogs had better cytotoxic effects toward a human colorectal cancer cell line HCT-15 compared to curcumin, suggesting cell apoptosis-inducing potential [19]. Apoptosis induction is an advantageous characteristic as an anticancer agent compared to necrosis induction because necrotic cells may cause inflammation that in turn leads to the drug conferring side effects upon administration [18]. In this study, the potential cytotoxic effects via apoptotic pathways using DK1 towards SW620 and HT29 colon cancer cells was assessed and the mechanism of apoptosis induction in the cell lines was further explored. The prominent difference between these two colon cancer cell lines is that HT29 cells were derived from primary colorectal cancer, which is less invasive by nature, while SW620 cells are highly metastatic. HT29 cells require a lower concentration of the chemotherapeutic agent 5-fluorouracil (5-FU) to decrease its viability by 50% compared to SW620 cells, which proves the SW620 cell line has a higher resistance towards anticancer agents than the HT29 cell line [20]. 

Results acquired from the MTT assay present the successful inhibition of cell proliferation in both HT29 and SW620 cells in a dose-dependent manner. As shown in Table 1, the overall inhibitory concentrations of DK1 and natural curcumin towards HT29 cells were lower than that of SW620 cells. This was probably due to the SW620 cells being more aggressive in nature than the HT29 cells. For the HT29 cells, natural curcumin required a concentration of 20.2 µM to exhibit 50% inhibition within 48 h while only 7.5 µM DK1 was needed to achieve a similar efficacy; approximately three times less than curcumin. Conversely for SW620 cells, only 14.5 µM of DK1 was needed to reduce the cell population by 50% while natural curcumin required more than 50 µM to achieve the same effect. These results showed that the curcumin analog DK1 is more potent than natural curcumin in inhibiting cell proliferation and inducing cell apoptosis. Previous studies have also indicated that natural curcumin required a concentration of 50 µM in48 h incubation time to achieve a 50% inhibitory concentration (IC_50_) inHT29 cells, whereas DK1 only needed 7.5 µM to exhibit a similar cytotoxic effect [21]. As for SW620 cells, a 96-h incubation time was needed to obtain a 50% inhibitory concentration (10 µM) compared to DK1 (14.5 µM), which required a slightly higher concentration but only 48 h to achieve the same cytotoxic effect [22]. Additionally, DK1 was also observed to be cytotoxic and could inhibit the proliferation of both cell lines, albeit a greater inhibition in HT29 cell proliferation compared to that of SW620. The AO/PI dual-staining assay was conducted to confirm that DK1 induces cell death via apoptosis by assessing the morphological changes between the untreated cells and the DK1-treated cells as depicted in Figure 2.Apart from the fluorescent signal given off by the cells, distinct features of apoptosis such as membrane blebbing, chromatin condensation, and cell shrinkage were observable. The AO stain can readily enter viable cells through the membrane pores due to its small molecular size and fluoresces green when observed using a fluorescent microscope; the PI stain is unable to enter the cell structure unless the cell membrane is compromised as a result from dying or dead cells [15]. Also, based on results from the annexin-V/FITC analysis in Figure 3, a population shift was observable in both cell lines. The externalization of phosphatidylserine occurs due to the disintegration of the membrane structure, indicating that DK1 induced cell death via apoptosis in both HT29 and SW620 cells. These results are also in agreement with previous studies conducted on breast cancer cells and osteosarcoma cells, which concluded that DK1 induces cell death through the apoptosis pathway [15,16].

To further assess the mode of cell death, the cell cycle activity was analyzed after the cells had been treated with the three concentrations (IC_25_, IC_50_, and IC_75_) of DK1. During a cell cycle there are checkpoints that play significant roles. They help to regulate the cell cycle by temporarily arresting the cell, thereby allowing time for cellular repair mechanisms to take place and reversing any incurred DNA mutation, or activating programmed cell death in the case of irreversible cellular damage and causing DNA fragmentation that is usually accompanied by increased sub-G0/G1 population in these apoptotic cells [17,23]. Irregular cell cycle activity then leads to enhanced cancer proliferation as the cell is unable to activate cell death mechanisms, thus allowing cancerous cells to further proliferate. In Figure 4, an increasing trend of the sub-G0/G1 population of HT29 cells was seen as the dose of DK1 increased. A similar trend was observed in SW620 cells where there was a significant accumulation of cells in the sub-G0/G1 phase. However, as the accumulation at the sub-G0/G1 phase was much higher in HT29 with a population of 74% compared to SW620 (subG0/G1 population of 23%), these results show that DK1 is more potent in inducing apoptosis inHT29 cells than in SW620 cells. The treatment with DK1 caused cell cycle arrest in both cell lines at the S phase, shown by the significant accumulation in the S phase and a significant decrease in the population at the G2/M phase.

Programmed cell death via apoptosis can be activated through two main pathways: the extrinsic pathway (activated by death receptors) and the intrinsic pathway (mitochondrial-dependent) [24,25]. When a stimulus such as a drug is administered, the mitochondrial membrane becomes permeabilized, leading to membrane rupture [18,26]. Apoptosis is mainly regulated by the caspase cascade [18]. When a cell undergoes apoptosis via the intrinsic pathway, the mitochondrial membrane depolarizes, becomes permeabilized, and starts to rupture, releasing pro-apoptotic factors such as cytochrome c and Smac, which leads to the formation of apoptosomes [18,27]. This then leads to the activation of a caspase cascade where, in this case, the activation of caspase-9 then activates effector caspases such as caspase-3, caspase-6, and caspase-7 [18]. On the other hand, when apoptosis occurs via an extrinsic pathway, the death receptors on the surface of the cell membranes are triggered and subsequently activate caspase-8, which will correspondingly activate effector caspases [18]. It was shown in Figure 5 that DK1 triggers depolarization of the mitochondrial membrane potential in both HT29 and SW620 cells, signifying that apoptosis occurrence was mitochondriadependent. However, concluding the mode of apoptosis solely based on the JC-1 assay is inadequate; therefore, several apoptotic-related genes and proteins were quantified in order to determine the mechanism of DK1 in inducing cell death.

As shown in Figure 6, caspase-3, caspase-9, and cytochrome C genes were significantly up-regulated when HT29 cells were treated with the IC_50_concentration of DK1 while in SW620 cells, caspase-8 and caspase-9 were significantly upregulated while caspase-3 was downregulated. These results suggest that both colon cancer cell lines underwent apoptosis via an intrinsic pathway upon treatment with DK1.The mechanistic activity of the curcumin analog DK1 was further examined using the human apoptosis proteome profiler. DK1 was able to upregulate the expression of several pro-apoptotic proteins (Table 2) such as pro-caspase-3, cleaved caspase-3, Bax, cytochrome C, HTRA2/Omi, and Smac/Diablo proteins. Bax is an extensively studied and widely known pro-apoptotic member of the Bcl-2 protein family of apoptosis-related proteins [27]. Bax regulates the mitochondrial membrane potential, which leads to the secretion of cytochrome C [18,24]. The secretion of cytochrome C induces the activation of the caspase cascade, leading to apoptosis [24]. HTRA2/Omi and Smac/Diablo proteins are also pro-apoptotic proteins that play significant roles as they help reduce the cell’s inhibition towards apoptosis [24,28,29]. However, upregulated expression of these pro-apoptotic proteinswere seen in the HT29 cells; in SW620 cells, only the HTRA2/Omi protein expression was upregulated and the other proteins did not show any significant difference in expression.This suggests that oxidative stress may partially contribute to the mitochondrial changes causing apoptosis in the SW620 cells [30]. The overall results show that DK1 is more potent to HT29 cells than SW620 cells, suggesting that it would potentially be a more effective anticancer agent for primary colon adenocarcinoma and more appropriate as an anticancer agent for treating colon cancer at early stages before the cancer metastasizes.

## 4. Materials and Methods

### 4.1. Synthesis of Curcumin Analogue, DK1

The curcumin analog DK1 used as treatment in this study was obtained from Dr. Nadeem Akhtar of Universiti Malaysia Pahang. DK1 was synthesized as outlined by Ali et al. [15].

### 4.2. Cell Culture

The HT29 cell line was maintained in Roswell Park Memorial Institute 1640 (RPMI-1640) medium while the SW620 cell line was maintained in Dulbecco’s Modified Eagle Medium (DMEM).Both media were supplemented with 10% fetal bovine serum and 1% penicillin–streptomycin (Life Technologies, Carlsbad, CA, USA) resulting in the complete growth media used to maintain the cell cultures. Cells were harvested to conduct assays as confluence reached 80% using TrypLE™ Express (Life Technologies, Carlsbad, CA, USA).Cell lines were maintained in culture flasks in 37°C humidified incubator provided with 5% CO_2_.

### 4.3. 3-(4,5-Dimethylthiazol-2-yl)-2,5-Diphenyltetrazolium Bromide(MTT) Assay

The MTT assay was conducted as outlined by Mosmann with slight modifications [31]. Briefly, each cell line was seeded in 96-well plates at a concentration of 5 × 10^4^ cells/mL. The cells were then incubated in a humidified CO_2_ incubator overnight to allow for cell attachment. The following day, natural curcumin and DK1 was added to the wells by two-fold serial dilution. The cell viability was measured at 24, 48, and 72 h posttreatment. The MTT reagent (Merck, Kenilworth, NJ, USA) was added at a volume of 20 µL of 5 mg/mL in each well and incubated for four hours. Afterwards, dimethyl sulfoxide (DMSO) was added to each well to solubilize the formazan crystals formed. The plates were then read using an ELISA plate reader at a wavelength of 570 nm (Bio-Tek Instruments, Winooski, VT, USA). The optical density values obtained were used to plot a dose–response curve. Triplicates were carried out for each cell line. The following formula was used to determine the percentage of viable cells.

(1) $$\mathrm{Percentage}\;\mathrm{of}\;\mathrm{Cell}\;\mathrm{Viability}\;=\frac{OD\;sample\;at\;570\;nm}{OD\;control\;at\;570\;nm}\times 100\%$$

The concentration of DK1 that reduced cell viability by 25%, 50%, and 75% compared to the untreated cells was called the inhibitory concentration (IC_25_, IC_50_, and IC_75_, respectively) and was determined by extrapolation from the dose–response curve of cell viability against treatment concentration. 

### 4.4. Cell Treatment

Based on the results of the MTT assay, three doses of DK1 (IC_25_, IC_50_, and IC_75_) were used for the following assays. The three doses—IC_25_, IC_50_, and IC_75_—of DK1 were administered to HT29 and SW620. As DK1 is water-insoluble, it was first dissolved in DMSO prior to mixing with complete growth media for treatment of the cells where the final DMSO volume administered to cells was less than 0.1%.

### 4.5. Acridine Orange/PropidiumIodide (AO/PI) Double-Staining Assay

AO/PI double staining was assayed with the aim of determining the morphological changes inHT29 and SW620 cells upon DK1 treatments. The cells were seeded in 6-well culture plates at a concentration of 3.0 × 10^5^ cells/well and incubated overnight. The next day, the cells were treated with the three concentrations (IC_25_, IC_50_, and IC_75_ values) of DK1 for 48 h. After incubation, the cells were detached and harvested. A 1:1 ratio of AO (10 µg/mL) toPI (10 µg/mL) was used to incubate the cells. The incubated cells were then viewed under a fluorescence microscope (Nikon, Tokyo, Japan) at 200× magnification with a filter range of 450–490 nm. 

### 4.6. Cell Cycle Analysis

The cells were seeded in 6-well culture plates at a concentration of 3.0 × 10^5^ cells/well and were incubated overnight. Three concentrations of DK1 were treated the following day. After 24 and 48 h, all cells were collected by trypsinization into phosphate buffer solution (PBS). The resulting pellet was fixed in 70% ethanol and stored at −20°C. After a week, the fixed cells were washed with PBS and processed using the Cycletest™ PLUS DNA Reagent Kit (BD Biosciences, San Jose, CA, USA) as outlined provided by the kit. Then, the cells were subjected to flow cytometric analysis using a NovoCyte flow cytometer (ACEA Biosciences Inc., San Diego, CA, USA).

### 4.7. Annexin-V/FITC Assay

The Annexin-V/FITC assay was carried out using the Annexin-V/FITC Apoptosis Detection Kit (BD Biosciences, San Jose, CA, USA). The cells were seeded in 6-well culture plates at a concentration of 3.0 × 10^5^ cells/well and were incubated overnight. The seeded cells were treated with the three concentrations (IC_25_, IC_50_,and IC_75_ values) of DK1 for 48 h. After the incubation times, the treated cells were all collected and harvested and the resulting pellets were immediately resuspended in the provided binding buffer and subsequently stained with 5 µL FITC annexin V and 5 µL PI. The mixture was left to incubate at room temperature for 15 min. Then, the cells were analyzed using a NovoCyte flow cytometer (ACEA Biosciences Inc., San Diego, CA, USA).

### 4.8. JC-1 (Mitoscreen) Assay

The BD™ Mitoscreen Mitochondria Membrane Potential Detection (JC-1) Kit (BD Biosciences, San Jose, CA, USA) was used to measure the depolarization of the mitochondrial membrane potential. The cells were seeded in 6-well culture plates at a concentration of 3.0 × 10^5^ cells/well and were incubated overnight. The seeded cells were treated with three concentrations (IC_25_, IC_50_,and IC_75_)of DK1. After 24 and 48 h, the cells were collected and harvested by trypsinization into PBS and then centrifuged at 2000 rpm for 5 min. The cells were incubated with 500 µL of JC-1 working solution. The JC-1 working solution contained the JC-1 stock solution and assay buffer at a ratio of 1:100. This mixture was incubated at 37 °C for 15 min. Finally, the cells were washed with the assay buffer twice before proceeding to the flow analysis using a NovoCyte flow cytometer (ACEA Biosciences Inc., San Diego, CA, USA).

### 4.9. Quantitative Real-Time PCR Assay 

Total RNA was isolated from the untreated and DK1-treated (IC_50_) cells using the QIAGEN RNeasy Kit (Qiagen, Hilden, NRW, Germany) according to the manufacturer’s protocol. The purity and concentration of the isolated RNA were measured using a Nanodrop™ spectrophotometer (Thermo Fisher Scientific Inc., San Diego, CA, USA) and the integrity of the RNA was determined by running a standard agarose gel. Then, 1 µg of the RNA was converted to cDNA using the RevertAid First Strand cDNA Synthesis Kit (Thermo Fisher Scientific Inc., San Diego, CA, USA) according to the manufacturer’s protocol. Afterwards, real-time PCR was carried out using the Maxima SYBR Green qPCR Master Mix (Thermo Fisher Scientific Inc., San Diego, CA, USA) on the Eco™ Real-Time PCR System (Illumina Inc., San Diego, CA, USA). The qRT-PCR regime included initiation at 95 °C for 10 s, and annealing/extension at 58 °C for 30 s. This process was repeated for 40 cycles and the results were analyzed using EcoStudy Software v.4.0. (San Diego, CA, USA) based on the efficiency of the primers. The results were analyzed to two housekeeping genes: ACTB and 18S rRNA. The fold change values were calculated by comparing the results of the untreated group and the DK1-treated group. The accession number and sequence of the selected genes are illustrated in Table 3.

### 4.10. Proteome Profiling

Total protein from HT29 and SW620 human colon cancer cell lines were extracted using 600 µL of radio immunoprecipitation assay (RIPA) buffer (50 mM Tris-HCl, 150 mMNaCl, 1.0% TritonX-100, 0.5% sodium deoxycholate and 0.1% SDS) and supplemented with 10 mg of protease inhibitor cocktail (Roche, Basel, Switzerland). The extracted protein was then quantified using Bradford assay (Sigma, St. Louis, MO, USA). The proteome profiling assay was conducted to confirm the induction of cell death via apoptosis and the expressed protein levels were determined using the Proteome Profiler™ Array Human Apoptosis Array Kit (R&D Systems, Inc., Minneapolis, MN, USA), conducted according to the manufacturer’s protocol provided in the kit. The membrane was scanned using the ChemiDoc XRS (BioRad, Hercules, CA, USA) and analyzed using Image Lab™ Software version 6.0.0 (Bio-Rad Laboratories, Inc., Hercules, CA, USA).

### 4.11. Statistical Analysis

All data presented with statistical mean ± standard deviation (SD) from three independent experiments using GraphPad Prism ver. 7.00 (GraphPad Software, Inc., La Jolla, CA, USA) was used to perform all statistical analysis. The statistical comparison analysis was done using the one-way analysis of variance (ANOVA), followed by Tukey’s *post hoc* test. The significance was set at *p* < 0.05. The comparisons done to obtain statistical significance was between the control (untreated) group and DK1-treated groups.

## 5. Conclusions

Overall, the curcumin analog DK1 was found to be more potent towards the less aggressive human colon cancer cell line, HT29, in inducing cell death via the intrinsic apoptotic pathway. This is supported by the upregulation of pro-apoptotic genes and proteins such as caspase-3, caspase-9, Bax, and cytochrome C. Further studies need to be conducted to assess the bioavailability potential of DK1 *in vivo* to confirm its efficacy as an anti-colon carcinoma drug.

## Figures and Tables

**Table 1:** The inhibitory concentrations of DK1 and natural curcumin in HT29 and SW620 colon carcinoma cell lines at 48 h posttreatment used as treatments in other assays. All data are expressed as the mean ± standard deviation (SD).

Cell Lines	Inhibitory Concentrations of DK1 (µM)			Inhibitory Concentrations (IC_50_) of Curcumin (µM)
	IC_25_	IC_50_	IC_75_	
HT29	2.8 ± 0.8	7.5 ± 1.6	28.0 ± 2.0	20.2 ± 0.6
SW620	3.7 ± 0.9	14.5 ± 1.2	27.8 ± 1.4	>50

DK1 = (*Z*)-3-hydroxy-1-(2-hydroxyphenyl)-3-phenylprop-2-ene-1-one.

**Table 2:** Apoptosis-related protein regulation in DK1-treated HT29 and SW620 cell lines.

Cells	Proteins	Relative Intensity (Fold Change)	Regulation
**HT29**	Pro-caspase-3	2.10 * ± 0.06	Up
	Cleaved caspase-3	1.98 ± 0.11	Up
	Bax	2.41 * ± 0.18	Up
	Cytochrome C	1.78 * ± 0.07	Up
	HTRA2/Omi	2.21 * ± 0.13	Up
	SMAC/Diablo	1.14 ± 0.01	Up
**SW620**	Pro-caspase-3	−0.98 ± 0.06	Down
	Cleaved caspase-3	−0.70 ± 0.05	Down
	Bax	−0.75 ± 0.04	Down
	Cytochrome c	−0.55 ± 0.02	Down
	HTRA2/Omi	1.25* ± 0.01	Up
	SMAC/Diablo	−0.96 ± 0.01	Down

Note: Human apoptosis proteome profiler of HT29 and SW620 colon cancer cell lines treated with DK1 (IC_50_) for 48 h. All data are expressed as mean ± SD. The (−) symbol indicates downregulated protein expression. * *p* < 0.05 compared with corresponding controls.

**Table 3:** The accession numbers and sequences of the primers used in the quantitative real-time PCR assay.

Gene	Accession No.	Forward Sequence(5′3′)	Reverse Sequence(5′3′)	Amplicon Size
*Caspase-3*	NM_004346	AGAACTGGACTGTGGCATTGAG	GCTTGTCGGCATACTGTTTCAG	191
*Caspase-8*	NM_001228	CATCCAGTCACTTTGCCAGA	GCATCTGTTTCCCCATGTTT	128
*Caspase-9*	NM_001229	TGTCCTACTCTACTTTCCCAGGTTTT	GTGAGCCCACTGCTCAAAGAT	101
*Bax*	BC014175.2	TTCTGACGGCAACTTCAACT	CAGCCCATGATGGTTCTGAT	153
*Cytochrome c*	NM_018947.5	GGGCCAAATCTCCATGGTCT	GGCAGTGGCCAATTATTACTC	246
*Cyclin A*	M25753.1	GGTGTCACTGCCATGTTTATTG	TCTGTCTGATTTGGTGCTTAGT	160
*CDK 2*	NG_029877.1	CCACAGCTACTCACCTGTTATC	ATGTCAACCCACCTTAATCTCTC	173
*ACTB*	NM_001101.3	AGAGCTACGAGCTGCCTGAC	AGCACTGTGTTGGCGTACAG	184
*18srRNA*	X03205	GTAACCCGTTGAACCCCATT	CCATCCAATCGGTAGTAGCG	151
